# Integrity of Induced Pluripotent Stem Cell (iPSC) Derived Megakaryocytes as Assessed by Genetic and Transcriptomic Analysis

**DOI:** 10.1371/journal.pone.0167794

**Published:** 2017-01-20

**Authors:** Kai Kammers, Margaret A. Taub, Ingo Ruczinski, Joshua Martin, Lisa R. Yanek, Alyssa Frazee, Yongxing Gao, Dixie Hoyle, Nauder Faraday, Diane M. Becker, Linzhao Cheng, Zack Z. Wang, Jeff T. Leek, Lewis C. Becker, Rasika A. Mathias

**Affiliations:** 1 Division of Biostatistics and Bioinformatics, Department of Oncology, Sidney Kimmel Comprehensive Cancer Center, Johns Hopkins University School of Medicine, Baltimore, Maryland, United States of America; 2 Department of Biostatistics, Johns Hopkins Bloomberg School of Public Health, Baltimore, Maryland, United States of America; 3 The GeneSTAR Research Program, Johns Hopkins School of Medicine, Baltimore, Maryland, United States of America; 4 Division of Hematology and Institute for Cell Engineering, Johns Hopkins School of Medicine, Baltimore, Maryland, United States of America; University of Minnesota Medical Center, UNITED STATES

## Abstract

Previously, we have described our feeder-free, xeno-free approach to generate megakaryocytes (MKs) in culture from human induced pluripotent stem cells (iPSCs). Here, we focus specifically on the integrity of these MKs using: (1) genotype discordance between parent cell DNA to iPSC cell DNA and onward to the differentiated MK DNA; (2) genomic structural integrity using copy number variation (CNV); and (3) transcriptomic signatures of the derived MK lines compared to the iPSC lines. We detected a very low rate of genotype discordance; estimates were 0.0001%-0.01%, well below the genotyping error rate for our assay (0.37%). No CNVs were generated in the iPSCs that were subsequently passed on to the MKs. Finally, we observed highly biologically relevant gene sets as being upregulated in MKs relative to the iPSCs: platelet activation, blood coagulation, megakaryocyte development, platelet formation, platelet degranulation, and platelet aggregation. These data strongly support the integrity of the derived MK lines.

## Introduction

Platelet aggregation on ruptured or eroded atherosclerotic plaques initiates arterial thrombosis and subsequently leads to acute ischemic syndromes such as myocardial infarction, stroke, and peripheral arterial occlusions [1]. We previously reported that platelet aggregation at baseline as well as after low dose aspirin are moderately to highly heritable [2] in both African Americans and European Americans. Using traditional genome-wide association approaches in families at increased risk for premature coronary artery disease (CAD) we successfully identified several common variants influencing platelet aggregation [3–6]. Cumulatively, these common variants account for only a fraction (<35%) of the total trait heritability observed in these families [2, 7]. Furthermore, all of these variants appear to be intronic or intergenic and their mechanism of action is not understood.

Despite major advances in our understanding of the potential regulatory role of non-coding DNA variants from a variety of consortia efforts, there is a lack of relevant ‘target’ tissue pertinent to platelet aggregation (i.e. platelets and megakaryocytes (MKs)) in these public catalogs [8, 9]. Pilot data by the GTEx (Genotype-Tissue Expression) project [10] on RNA sequencing data from 1641 samples across 43 tissues confirms that the specificity or commonality of expression quantitative trait loci (eQTLs) among tissues and cell types [11, 12] yields valuable insights into differential genetic regulation among tissues that is of biological significance. With respect to the platelet aggregation phenotype, platelets are by themselves anucleate cells that have a limited life span (7–10 days) and capacity for de novo protein translation [13]. They are generated from bone marrow MKs from which they obtain messenger RNA. Therefore, MKs are a critical ‘target tissue’ relevant to our interrogation of platelet aggregation because they are relevant to understanding the regulation of transcript and protein levels ultimately observed in platelets.

Megakaryocytes are difficult to obtain in sufficient numbers from large numbers of subjects because they reside in bone marrow that is only available by invasive bone marrow sampling techniques. Furthermore, the level of megakaryocytes is exceedingly low (<0.01%) in bone marrow [14] increasing the difficulty in accessing this specific target cell type with traditional RNA sequencing approaches. This is a major limitation in the analysis of large numbers of study subjects with platelet relevant phenotypes such as platelet aggregation and responsiveness to aspirin therapy where the target tissue includes platelets and the precursor megakaryocyte. To overcome this barrier, we generated induced pluripotent stem cells (iPSCs) from peripheral blood mononuclear cells on N = 250 subjects and subsequently differentiated these iPSCs into MKs. The details of the experimental approach for producing MKs from iPSCs have been described in detail elsewhere [15]. In this work we extend the prior analysis of the iPSC-derived MKs looking specifically at the integrity of the derived cells from a genetic (structural variation in the genome of the iPSC and MK relative to the donor MNC) and transcriptomic perspective (looking at gene set enrichment accounting for the differential expression by direction between the iPSC and MKs). In future work, these MKs will serve as a substrate through which we hope to better understand the genetic determinants of transcript regulation in MKs and ultimately understand the genetic determinants of platelet aggregation.

## Results

### Genotype Integrity of iPSC Derived MKs

**Fig 1:**
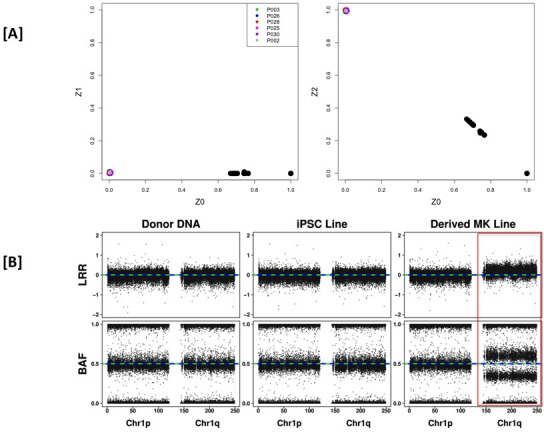
Genetic and genomic structural integrity of iPSC derived MK cells. **[A]** IBD analysis for all cell line pairs across 6 subjects and **[B]** CNV analysis showing a full duplication of the long arm of chromosome 1 in one MK cell line (MP030A). In Panel A pairs representing DNA from donor MNC, iPSC or MKs within a single subject are represented in color, pairs representing DNA from donor MNC, iPSC or MKs between subjects are represented in black and Z0,Z1,Z2 = probability of sharing 0,1 and 2 alleles IBD, respectively. In Panel B, LRR = Log R Ratio and BAF = B Allele Frequency. The full duplication of the long arm of chromosome 1 is highlighted in the red box.

The identity by descent (IBD) analysis (**Fig 1A**) indicates that all pairs of cell lines (donor MNC, iPSC and MK pairs as summarized in S1 Table) *within* any single subject have a Z2≈1 and a Z1≈Z0≈0 where Z2, Z1 and Z0 are the probabilities of sharing 2, 1, and 0 alleles identical by descent between the pair, respectively. This suggests that all DNA samples within a single subject are essentially identical. All DNA samples *between* subjects have Z2 <<1 suggesting essentially an ‘unrelated’ status.

The direct genotype concordance estimates between pairs of cell types within a subject (**S2 Table**) indicate a very low rate of discordance; estimates range from 0.0001%-0.01% which are far below the genotyping error rate (0.37%), and are most likely due to genotyping error. As an additional measure, we evaluated the occurrence of a discordancy from the original iPSC line that may have then been passed from the iPSC to the derived MK cells (**S2 Table**). Here too, rates are very low (0–8 discordant genotypes were passed from original iPSC➜MK). Assuming that these ‘transmitted’ discordant genotypes reflect a true mutation and not a genotyping error (because they appear to have occurred in the iPSC and then seem to pass along to the resulting MK), we estimated this ‘mutation rate’ to be 0–0.0008% or ~1 in 10^−6^ which appears to be within the normal expected mutation rate occurring during cell division. We should highlight the caveat that the discordancy may be due to a genotyping error in the parent MNC, and therefore while our estimates may be upwardly biased they are still well within expectation.

### Genomic Structural Integrity of the iPSC Derived MKs

On average we observe 3.0 copy number variation (CNV) differences between the paired donor MNC➜iPSC (i.e. CNV not called in the MNC but called in the iPSC), and 1.3 CNV differences between paired iPSC➜MK (i.e. CNV not called in the iPSC but called in the MK). However, all of these observed differences appear to have been due to false positive identifications in the transformed cells, or false negative identifications in pre-transformed cells (see **S1** and **S2 Figs**). Any CNVs observed in the original donor DNA were also observed in the respective iPSCs and MKs (see **S3 Fig**). The only exception was a real CNV in an MK line that was not present in parent DNA or iPSC DNA: a complete duplication of the long arm of chromosome 1 (**Fig 1B**) that was detectable and replicated by the RNA-sequencing data below.

### Transcriptomic Integrity of the iPSC derived MKs

#### Overview of RNA-sequencing data

**Fig 2:**
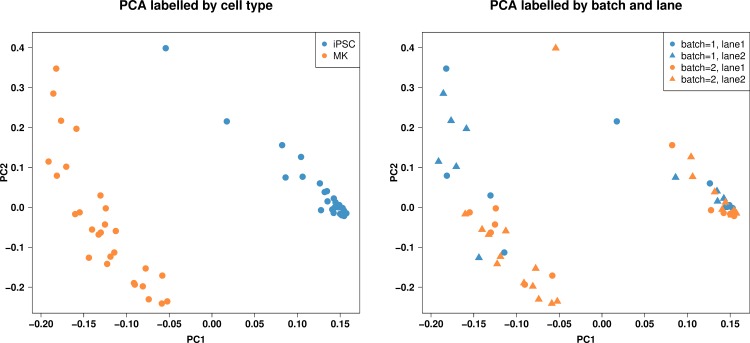
Principal component analysis (PCA). The principal component (PC) score plots show the relationship between cell type (induced pluripotent stem cell (iPSC) or megakaryocyte (MK), left panel), and batch and lane (right panel) in terms of PC1 (x-axis) and PC2 (y-axis) from a PCA of 33,287 transcripts with FPKM interquartile range larger than 1. Cell type is highly associated with PC1 and thus cell type explains most of the expression variation in this data set. The scores of the first two PCs do not show apparent patterns for batch and lane.

We detected 782,988 assembled transcripts in the 56 total RNA-sequencing datasets of which we retained 33,287 transcripts with FPKM interquartile range across all 56 RNA-sequencing samples larger than 1. To obtain an overview of the data structure of the 56 RNA-sequencing data sets, the raw data was filtered as described above and used for a principal component analysis (PCA). Visualization of the data along the first two principal components (PC) indicated two distinct clusters. Labeling the PC scores by cell type (Fig 2, left panel) reveals that cell type (iPSC vs MK) is highly associated with this first PC and thus explains most of the expression variation in this data set. We observe here that variability within the 28 iPSC samples is considerably lower than that within the 28 MK samples. The scores of the first two PCs do not show apparent patterns for sequencing batch or lane (Fig 2, right panel).

### Differential Expression

**Fig 3:**
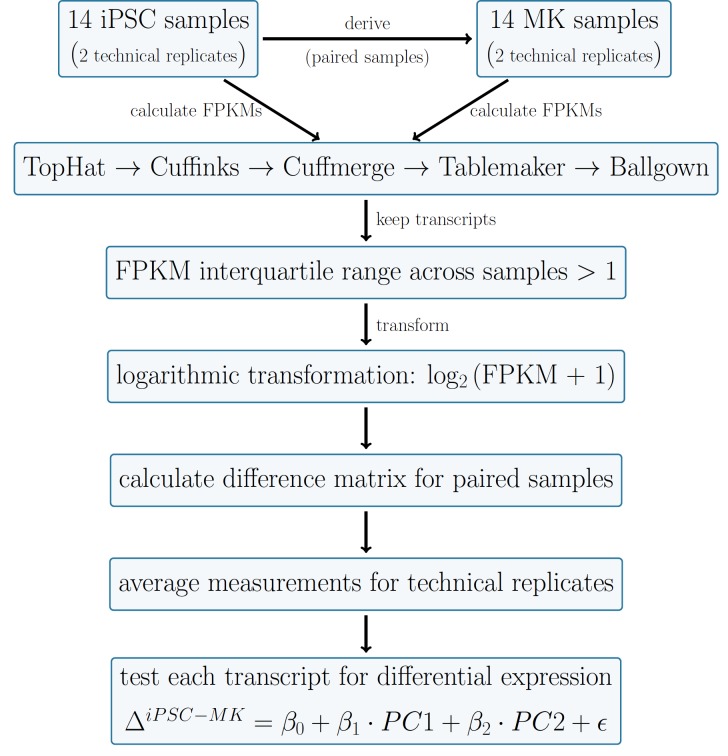
RNA-sequencing analysis pipeline. Reads of 28 iPSC samples and 28 MK samples (each 14 subjects with 2 technical replicates) were analyzed using the standard steps in the Tuxedo pipeline until the Cuffmerge step, after which Tablemaker was used to calculate per sample FPKMs. These results were loaded into R using the Ballgown package. Transcripts with FPKM interquartile range smaller than 1 were excluded. After logarithmic transformation of the transcript expression data set, differences between paired iPSC and MK samples were calculated and measurements were averaged for technical replicates. Differences in transcript expression between MK and iPSC samples was measured in a linear model framework with adjustments for the first two principal components of the expression data.

To account for the fact that each of 14 subjects included in the transcriptomic analysis (see **S1 Table**) has paired iPSC-MK measurements, and that each iPSC and MK sample has two technical replicates (referred to as A and B lines), the outcome variable for differential expression analysis for each of the N = 14 subjects was the average of their two expression differences (average of iPSCA-MKA and iPSCB-MKB for paired samples). This is defined in detail in **Fig 3**. For each transcript, we tested whether the mean of the expression differences across 14 subjects accounting for the technical duplicates was statistically different from zero. PCA analysis of the data set reveals that the first two PCs account for 47.1% and 13.0% of the variation, respectively (**S4 Fig**). Since the subject size of 14 is relatively small and adjusting for several covariates would lead to a decrease in power to detect differentially expressed transcripts, we adjusted for the first two principal components and used moderated t-statistics for inference (see Eq (1)). Since the first two principal components account for a large fraction of the variability in the data, adjusting for PCs rather than explicitly adjusting for traditional confounders like age, sex and batch, passage number, as well as the percent CD41+CD42a+ megakaryoblasts in the MK cell pellet, allowed us to control for potential confounders while keeping the reduction in power to detect our signal of interest at a minimum. That is, if one of these confounding factors contributes to a large fraction of the variability of our data (and hence could have a large effect on the outcome measurement of interest) it will be controlled for by including the PCs in the model, while if the factor does not contribute to variability in the data, we do not pay a penalty in power by including it in the linear model unnecessarily.

For this differential expression analysis we consider directionality of transcript differences between iPSC and MK cell lines. Among the 33,287 transcripts, we identified 15,284 transcripts that were statistically significantly up-regulated in MKs compared to iPSCs and 17,555 transcripts that were statistically significantly down-regulated at a false discovery rate (FDR) of 5%. A volcano plot and histograms of p-values separated by directionality of the fold change between iPSCs and MKs illustrate the large number of differentially expressed transcripts (**S5 Fig**). Full sets of gene lists that were differentially expressed are presented in **S3** and **S4 Tables** and are available for download.

### Gene set Enrichment

We performed pathway analysis using gene set enrichment analysis, first splitting the differential expression results into two subsets depending on the directionality of transcript differences between iPSC and MK cell lines. Using the ‘biological process’ ontology, we detected 94 gene sets at an FDR of 5% (q < 0.05) showing enrichment for genes that were up-regulated in MKs compared to iPSCs (**Table 1** lists those with a q < 0.001). Among these 94, we identified the following biologically relevant gene sets: “platelet activation” (GO:0030168), “inflammatory response” (GO:0006954), “megakaryocyte development” (GO:0035855), “platelet formation” (GO:0030220), “platelet degranulation” (GO:0002576), “platelet aggregation” (GO:0070527), “regulation of cell proliferation” (GO:0042127), and “immune response” (GO:0006955). At a FDR threshold of 5%, we identified 15 gene sets that are enriched among genes that were down-regulated in MKs compared to iPSCs (**Table 2**).

**Table 1:** Significant GO groups for transcripts with higher expression in MKs compared to iPSCs.

GO.ID	Term	Annotated	Significant	Expected	p-value	q-value
**GO:0008150**	biological_process	6357	6301	6256.06	<0.0001	<0.0001
**GO:0008152**	metabolic process	4998	4950	4918.64	<0.0001	<0.0001
**GO:0007165**	signal transduction	2262	2242	2226.08	<0.0001	<0.0001
**GO:0030168**	platelet activation	172	172	169.27	<0.0001	<0.0001
**GO:0045087**	innate immune response	566	559	557.01	<0.0001	<0.0001
**GO:0007596**	blood coagulation	329	326	323.78	<0.0001	<0.0001
**GO:0007229**	integrin-mediated signaling pathway	51	51	50.19	<0.0001	<0.0001
**GO:0044267**	cellular protein metabolic process	2440	2409	2401.25	<0.0001	<0.0001
**GO:0006954**	inflammatory response	277	275	272.6	<0.0001	<0.0001
**GO:0035855**	megakaryocyte development	15	15	14.76	<0.0001	<0.0001
**GO:0007275**	multicellular organism development	1887	1871	1857.04	<0.0001	<0.0001
**GO:0030220**	platelet formation	18	18	17.71	<0.0001	<0.0001
**GO:0045665**	negative regulation of neuron differentiation	71	70	69.87	<0.0001	<0.0001
**GO:0002576**	platelet degranulation	57	57	56.09	<0.0001	0.0001
**GO:0070527**	platelet aggregation	40	40	39.36	<0.0001	0.0002
**GO:0042127**	regulation of cell proliferation	645	639	634.76	<0.0001	0.0002
**GO:0060216**	definitive hemopoiesis	16	16	15.75	<0.0001	0.0002
**GO:0006810**	transport	2006	1995	1974.15	<0.0001	0.0002
**GO:0035556**	intracellular signal transduction	1304	1288	1283.29	<0.0001	0.0005
**GO:0007186**	G-protein coupled receptor signaling pathway	198	196	194.86	<0.0001	0.0006
**GO:0030154**	cell differentiation	1472	1459	1448.63	<0.0001	0.0008
**GO:0032496**	response to lipopolysaccharide	164	164	161.4	<0.0001	0.0009

Only GO groups with q < 0.001 are presented. The table includes the GO identifier “GO.id”, the gene ontology category name “Term”, and the number of annotated, significant, and expected genes. “p-value” (based on a Kolmogorov-Smirnov test) and “q-value” show the statistical significance of enrichment before and after correction for multiple comparisons, respectively.

**Table 2:** Significant GO groups for transcripts with lower expression in MKs compared to iPSCs.

GO.ID	Term	Annotated	Significant	Expected	p-value	q-value
**GO:0008150**	biological_process	7955	7907	7867.93	<0.0001	<0.0001
**GO:0008152**	metabolic process	6263	6223	6194.45	<0.0001	<0.0001
**GO:0006355**	regulation of transcription, DNA-templated	1825	1816	1805.02	<0.0001	<0.0001
**GO:0007275**	multicellular organism development	2332	2321	2306.48	<0.0001	<0.0001
**GO:0007156**	homophilic cell adhesion via plasma membrane adhesion mol.	49	49	48.46	<0.0001	<0.0001
**GO:0007165**	signal transduction	2449	2435	2422.2	<0.0001	<0.0001
**GO:0034641**	cellular nitrogen compound metabolic process	3744	3724	3703.02	<0.0001	<0.0001
**GO:0044267**	cellular protein metabolic process	2889	2869	2857.38	<0.0001	<0.0001
**GO:0016477**	cell migration	532	529	526.18	<0.0001	0.0007

Only GO groups with q < 0.001 are presented. The table includes the GO identifier “GO.id”, the gene ontology category name “Term”, and the number of annotated, significant, and expected genes. “p-value” (based on a Kolmogorov-Smirnov test) and “q-value” show the statistical significance of enrichment before and after correction for multiple comparisons, respectively.

### RNA-Sequencing Validation of CNVs Found

**Fig 4:**
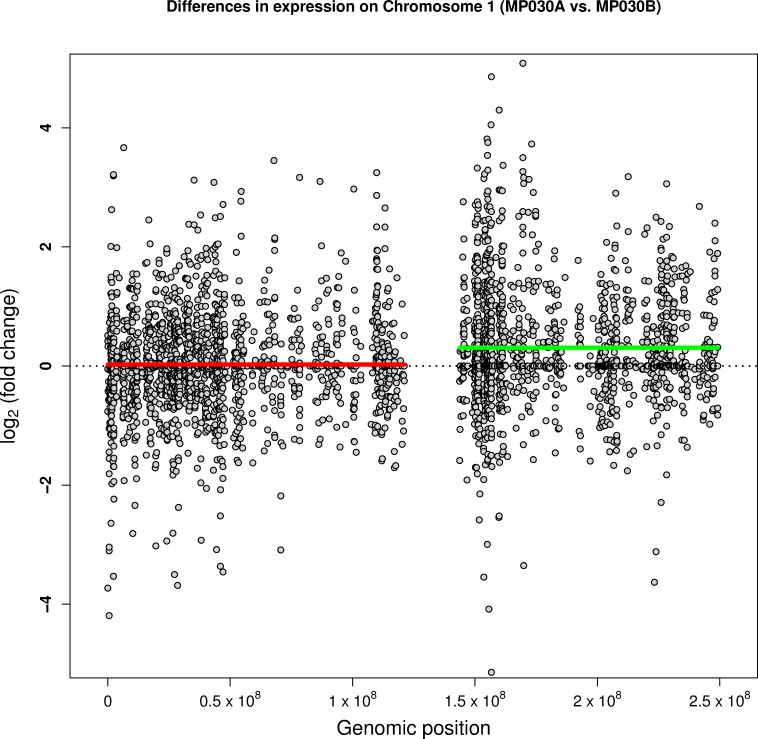
**Plot of fold change of chromosome 1 transcripts comparing the two MK technical replicates A and B from subject MP030.** Technical replicate A has an identified duplication of the q-arm of chromosome 1. The x-axis represents the genomic position of the transcript and the y-axis represents the log_2_ fold change between technical replicates A and B. Each point indicates the log_2_ fold change for one transcript. The green line indicates the mean log_2_ fold change for transcripts of the q-arm, highlighting the fact that transcripts in technical replicate A are more highly expressed than in technical replicate B. The mean log_2_ fold change for transcripts of the p-arm (red) is drawn as reference. The horizontal dotted black line indicates zero differences between the two technical replicates.

We observed a duplication of the q-arm of chromosome 1 for technical replicate “A” of subject MP030 in the CNV analysis described above. As a novel extension to our analysis approaches to CNV detection, we investigated first if the large duplication could be detected using the transcriptome data and second, if this duplication affected the downstream GO results derived from observed differential transcript expression. To this end, we first investigated transcript expression for the two MK lines A and B of subject MP030, where only the A line had the aberrant duplication but the B line appeared normal as compared to the parent DNA from the MNC. **Fig 4** shows log_2_ fold change between the two lines versus the genomic location. The green line indicates the mean log_2_ fold change for transcripts of the q-arm, highlighting the fact that transcripts in technical replicate A are more highly expressed than in technical replicate B, although we note that the mean fold-change is well below 2. The mean log_2_ fold change for transcripts of the p-arm (red) is drawn as reference. We also conducted a sensitivity analysis, in which we excluded replicate A of subject MP030 (and its corresponding iPSC partner) and performed differential expression and GO analysis. The results showed that the overall influence of the duplication of the q-arm chromosome in one sample is very small. In particular, the lists of highly significant GO groups presented in **Table 1** contained the same GO groups when excluding the designated sample. This is likely because the magnitude of the differential expression comparing the iPSC to MK samples is much larger than the magnitude of the expression difference in the individual carrying the duplication. The end result is that even in the presence of a large-scale genetic change, the integrity of the iPSC-MK comparison signal remains intact.

## Discussion

Genomic instability in human pluripotent stem cells (hPSCs) has been recognized since the early 2000s [16, 17]. The first reports included large-scale genomic differences with karyotypic abnormalities including trisomies [16, 18] and more recently, sub-chromosomal abnormalities such as gene duplications/deletions and point mutations [19–21]. Similar to hPSCs, induced pluripotent stem cells (iPSCs) also appear to suffer from similar genomic instabilities [19, 22, 23]. Of note, iPSCs appear to have considerably higher numbers of sub-chromosomal copy number variants (CNVs) compared to hPSCs [24–26]. Prior studies have shown variations in genomic instability as a function of the cell engineering technology applied [27] (i.e. choice of reprogramming methods).

In this study we document the high integrity of the MK lines generated from iPSCs in our previously described feeder-free, xeno-free approach [15]. In the prior work [15], our analysis on the integrity of the iPSC and derived MKs was limited to differential transcriptomic analysis at the single-gene level. Here, we expanded those analyses to look at integrity from a genetic perspective including structural variation and we extend the transcriptomic work to include gene set enrichment analysis on the sets of genes differentially expressed.

Using genotype data from paired iPSC and MK lines and comparing to the parent mononuclear cell DNA we show very low levels of genotype discordance between the paired lines. Levels of genotype discordance noted are within the expectations of somatic mutation rates. With the exception of the complete duplication of chromosome 1q in one MK line, we also observed remarkable genomic structural integrity of the iPSCs and iPSC derived MKs. Any detected CNV differences between the cell lines (donor MNC, iPSC and MK) were identified to be false positives upon manual examination of the called CNV as documented in **S1** and **S2 Figs**. For the manual inspection, the log R ratio (LRR) and B allele frequency (BAF) plots were visually contrasted between the parent DNA, iPSC and MK to inspect the differences. Inferring deletions and amplifications based on LRRs and BAFs using a hidden Markov model, we observed that CNVs in the original donor DNA were also present in the respective daughter cells. We also specifically investigated the possibility of an introduction of structural variation in the pairings of donor DNA to iPSC line, and iPSC line to MK line. We were able to manually inspect all inferred CNVs in a daughter cell but not inferred in the respective parent cell, as the total number of such instances was very low. For each pair, the LRRs and BAFs were qualitatively the same, indicating either a false positive identification in the daughter cell or a false negative in the parent cell. False positives and false negatives are common occurrences when inferring CNVs from SNP data, as the CNV calls are mostly based on the observed total allele intensities, a much more technically variable quantity for example compared to the relative allele intensities that genotype calls are based on; this technical variability results in more difficulty in calling CNVs as compared to SNPs [28, 29].

We extended the investigation of the integrity of our MK lines to the transcript level in these analyses. We observed clear differences in clustering based on PCs between the two cell types as expected with lower variability between iPSCs than between MKs. There were large numbers of transcripts that were significantly different between the two cell types and we therefore focused on gene set enrichment analyses to identify sets of genes that distinguish the two types. To effectively do this, we applied a novel approach of direction-specific differences between the MK and iPSC lines, i.e. sub-setting all differentially expressed genes into two separate classes: those up-regulated in MKs compared to iPSC and those down-regulated in MKs compared to iPSC lines. The sets of genes enriched differed between these two subsets. Many of the most significantly enriched gene sets pertained specifically to platelet function and development: “platelet activation” (GO:0030168), “inflammatory response” (GO:0006954), “megakaryocyte development” (GO:0035855), “platelet formation” (GO:0030220), “platelet degranulation” (GO:0002576), “platelet aggregation” (GO:0070527), “regulation of cell proliferation” (GO:0042127), and “immune response” (GO:0006955). These patterns and the sets of genes upregulated in the MKs as compared to the iPSC help to confirm the integrity of the derived cell type.

One of the variables of relevance in the derivation of the MKs from the iPSCs is the percent CD41+CD42a+ megakaryoblasts in the MK cell pellet ultimately used in the transcriptomic analysis presented here. We observed a range of 41% to 94% across the 28 MK RNA-sequencing experiments (14 subjects each with an A and B derived MK line). Our principal components analysis (**S6 Fig**) does not support any differences in the divergence of the MK lines from the iPSC lines based on their percentage of CD41+CD42a+ megakaryoblasts in the MK cell pellet.

With respect to some of the analytical aspects of our analysis, we have shown that a technical issue that arises in the comparison of our iPSC to MKs lines was the high number of transcripts that were not expressed in the iPSCs but on the other hand were expressed in the MKs at low-modest levels. Traditional RNA-sequencing quality control filters such as the mean and variance filters would have resulted in these transcripts being excluded from our investigations; i.e. transcripts highly relevant to our assessment of MK integrity would have been dropped prior to analysis. To accommodate this we implemented the novel approach of the interquartile range (IQR) filter and have shown in this analysis how it accommodates these transcripts successfully (**S7 Fig**). Another analytical contribution in this work is the capture of a large CNV (here the entire duplication of the q arm of chromosome 1) in a single sample relying solely on RNA-sequencing data. This approach will offer us the unique ability to assess large CNVs in all our MK cell lines (ultimately up to 250 GeneSTAR subjects) utilizing RNA-sequencing data alone even in the absence of genotype array data or karyotyping.

In conclusion, this work shows that megakaryocytes (MKs) differentiated from induced pluripotent stem cells (iPSCs) appear to have high integrity retaining their genetic architecture and developing a strong MK signal through the process. While we recognize that it would have been ideal to have megakaryocytes from bone marrow of the study subjects as a comparison to the iPSC-derived MKs, this is not feasible because they reside in very low levels (<0.01%) in bone marrow [14] and furthermore, are available only by invasive bone marrow sampling techniques. Therefore, through the generation of iPSC-derived MKs and the downstream analysis integrating genetics, transcriptomics and eventually epigenetics with methylation patterns, we hope to better understand the genetic determinants of transcript regulation in MKs and ultimately understand the genetic determinants of platelet aggregation. To this end, our analyses herein document integrity of these derived MKs, and confirm a transcriptomic signature that appears to be highly reflective of MK biology.

## Material and Methods

### Study Participants

Participants were recruited from our established GeneSTAR study [2, 3, 30]. Briefly, they came from European American (EA) and African American (AA) families with a history of premature CAD (onset <60 years). Healthy family members of affected probands were eligible if they were free of clinically apparent atherosclerotic disease or any other serious comorbidity. N = 15 subjects (N = 11 African American and N = 4 European American) were selected to be included in the pilot phase of the study in which iPSCs were reprogrammed from MNCs, and MKs were derived from the iPSCs (see Supplementary **S1 Table**). The study was approved by the Johns Hopkins Medicine Institutional Review Board and all participants provided written informed consent. In accordance with the consents signed by the GeneSTAR subjects, our data are deposited into dbGaP (phs001074.v1.p1) for access.

### Sample and Subject Labeling Nomenclature

Supplementary **S1 Table** presents a detailed overview of the 15 subjects included in this study, the cell lines available on each subject, and the specific data (genotype and/or transcriptome) available on each. Each independent subject is represented by a ***PXXX*** label. The prefixes of ***I*** and ***M*** represent the iPSC and MK from that subject, respectively. The suffixes ***A*** and ***B*** represent the two alternate lines generated on each subject; these represent technical replicates. For example IP002A and MP002A represent the iPSC line A for individual P002 and the MK derived from iPSC line A for individual P002, respectively. Similarly, IP002B and MP002B represent the iPSC line B for individual P002 and the MK derived from iPSC line B for individual P002, respectively. There are a total of 15 independent subjects represented in the data; 6 of these are included in the genotyping arm of this study (see **S1 Table** for details) and 14 are included in the RNA-sequencing arm (see **S1 Table** for details, also referenced in **Fig 2**).

### Generation of iPSC and Derived MKs

The protocols used to generate the iPSC and derive MKs are described in detail by Liu et al. [15]. Briefly, iPSC lines were reprogrammed from peripheral blood MNCs using non-integrating episomal vectors. After establishment, they were all expanded in Essential 8 medium on either Matrigel (1:30; BD Biosciences, San Diego, CA, http://www.bdbiosciences.com) or vitronectin (5 μg/cm^2^, Life Technologies). Human iPSCs were differentiated into definitive CD34+CD45+ hematopoietic progenitor cells (HPCs), using the “spin-embryoid body” (spin-EB) method in feeder- and serum-free conditions. Single iPSCs were suspended in serum-free medium (SFM). On day 14, the suspended cells were harvested and seeded for MK culture, generating a cell population enriched for CD41+CD42a+ megakaryoblasts. We used Food and Drug Administration (FDA)-approved pharmacological agents to replace thrombopoietin (TPO) and bovine serum albumin (BSA) in the culture medium, an important factor for future clinical applications[15]. There is variability in the passage number of our iPSC (see **S1 Table**) which is accounted for in the transcriptomic analysis using principal components as covariates in the models of analysis as described below.

### Genotyping Protocol, Genotype Calling, Data Quality and CNV Analysis

Genotyping was performed at the Center for Inherited Disease Research at Johns Hopkins using the HumanOmniExpressExome-8v1 array on DNA from 6 different subjects. For each, we genotyped the donor MNCs and up to two iPSC and paired derived MK lines per subject. A total of 28 DNA samples were run including 4 HapMap CEPH controls and DNA from 6 independent GeneSTAR subjects (see **S1** and **S2 Tables**). Genotype calls were made using GenomeStudio version 2011.1 and Genotyping Module version 1.9.4 and a total of 946,674 SNPs (99.53% of attempted SNPs) were released. The genotyping error rate based on the 4 HapMap CEPH samples was determined to be 0.37%.

To test for genotype integrity three different analyses were performed. First genotype data were read into PLINK [31] for all 24 samples and the amount of DNA shared IBD was estimated across all 24 samples using a set of linkage disequilibrium (LD) pruned markers. Markers were pruned using a window size of 50, step of 5 and r^2^ threshold of 0.3. Second, we calculated the actual genotype mismatches between each pair of lines MNC➜iPSC and iPSC➜MK and determined the number of discordances between each pair. Finally we examined how many mismatches between the MNC➜iPSC were also passed on the to MK line; we use this count as the estimation of a mutation rate in the iPSC reprogramming. However, we acknowledge that this could also include instances where there is a genotyping error in the MNC (i.e. if the MNC were genotyped incorrectly at a single SNP, then in comparing the SNP call in the MNC, iPSC and MK, it would appear as a mutation in the MNC➜iPSC that was also passed on the to MK line).

The LRRs (a normalized measure of the total signal intensity for two alleles of the SNP) and the BAFs (a normalized measure of the allelic intensity ratio of two alleles) were available on the 946,674 genotyped SNPs. CNVs were called using a hidden Markov model developed by investigators on our team [32] using a threshold of 10 supporting probes to call deletions and amplifications. We investigated in particular the possibility of systematic introduction of structural variation in the pairings of (i) donor DNA to iPSC line and (ii) iPSC line to MK line. As with the genotype integrity, we were specifically interested in CNVs that arose in the iPSC line that were then transferred down to the derived MK line. Any candidate CNVs were then visually inspected across matching DNA samples to rule out the possibility of false-positive calls (see **S1**–**S3 Figs**).

### RNA-Sequencing Data

We performed RNA-sequencing on non-ribosomal mRNA derived from iPSC and MK cell pellets from 14 subjects each with two technical replicates of paired iPSC and MK cell lines (the A and B lines). The percent of CD41+CD42a megakaryoblasts in the MK cell pellet was determined prior to RNA extraction. A flowchart representing the RNA-sequencing pipeline is shown in **Fig 3**.

### RNA-Sequencing Data Preprocessing

For alignment and assembly we used the Tuxedo pipeline [33]. Reads were aligned to human genome (UCSC, hg19) using the spliced-read mapper TopHat2 (version 2.0.13; [34]). Transcripts were assembled with Cufflinks, and finally merged with Cuffmerge using the UCSC reference annotation genes.gtf (version archive-2014-06-02-13-47-56) to guide reference annotation based transcript assembly (“-g” argument in the software, version 2.2.1; [33]). The output included all reference transcripts as well as novel genes and isoforms that were assembled. We used Tablemaker[35] (version 2.1.1) to estimate FPKM (fragments per kilobase of transcript per million reads sequenced) for each assembled transcript. In order to perform statistical downstream analysis we integrated the results from Tablemaker into the software environment R (version 3.3.0; [36]) with the R-package Ballgown (version 2.4.2; [35]).

### Differential Expression

Differential expression analysis was carried out at a transcript level. We filtered transcripts with an FPKM interquartile range (IQR) across all 56 RNA-sequencing experiments greater than 1. We chose an IQR filter because: (1) we found it to be robust to outliers as in the case of transcripts only expressed highly in one sample but not in any others, that may not be excluded by a mean or variance-based filter (**S7 Fig**, Transcript 4); and (2) it was sensitive to the two different cell types included in the design of our study, where a transcript could be essentially absent in one cell type but present, even at a low level of expression, in the other (**S7 Fig**, Transcript 3).

The FPKM values of the filtered transcripts were log-transformed using log_2_ (FPKM + offset). We added an offset = 1 to the FPKM values before log_2_ transformation to facilitate calculation. To incorporate the paired design of our data, we first calculated paired differences of log-transformed FPKMs by transcript within each line A or B (i.e. the differences in transcript expression of iPSCA-MKA and iPSCB-MKB). We then averaged expression values of these two technical replicates. This averaged data set is denoted by Δ^*iPSC*−*MK*^. In general, for each transcript we tested the null hypothesis that the differences in the pairs of observations came from a normal distribution with mean equal to zero and unknown variance, using the one-sample t-test. In order to adjust for potential confounding factors, we adjusted for the two principal components of the observed data set containing paired differences of transcript expression. The following linear model was fit for every transcript:

$$\Delta_{ij}^{iPSC-MK}=\beta_{0,i}+\beta_{1,i}∙{PC1}_{j}+\beta_{2,i}∙{PC2}_{j}+\epsilon_{ij}$$ (1)

where $\Delta_{ij}^{iPSC-MK}$ denotes the differences in the iPSC-MK pairs of log-transformed FPKM for transcript *i* in sample *j* and *ϵ*_*ij*_ the error term. *PC*1 and *PC*2 are the scores of the first and second principal components, respectively, calculated from Δ^*iPSC*−*MK*^. The parameter of interest for differential expression analysis of transcript *i* is the intercept *β*_0,*i*_.

Differential expression was calculated using the R package limma (version 3.28.5, [37]). Here, the observed transcript sample variances were shrunk towards a pooled variance estimate in order to obtain more stable variability estimates [38]. This resulted in moderated test statistics and thus moderated p-values. For multiple comparison correction we calculated q-values from the observed moderated p-values [39]. If a transcript has a q-value of 0.05, we expect 5% of the transcripts that show smaller p-values to be false positives, i.e., the q-value controls the expected FDR at 5%. Transcripts with calculated q-values smaller than 0.05 between iPSCs and MKs were declared statistically significant.

### Gene Set Enrichment

Gene Ontology [40, 41] group enrichment analysis was performed using the R package topGO (version 2.24.0, [42]), and results from the *biological process* ontology were reported. We used the default algorithm *weight01* in combination with the Kolmogorov-Smirnov test to assess gene group enrichment.

## Supporting Information

S1 Table Overview of study subjects. Sources of DNA and RNA by cell type, passage number of iPSC lines and percent CD41^+^CD42a^+^ megakaryoblasts in MK pellets by study subject.
(PDF)

S2 Table Genotype discordances. Counts of discordant genotypes out of a total of 946,674 SNPs between pairs of lines within 6 GeneSTAR subjects. All samples had genotype data on DNA from the single donor MNCs, and the first line (A line) of iPSC with its corresponding derived MK. Five samples had a second line (B line) of iPSC and one sample also had the corresponding MK. Transmitted discordancies represent the discordant genotypes that are observed in the donor MNC-iPSC that is noted to be transmitted on to the corresponding derived MK.
(PDF)

S3 Table List of transcripts for which MK expression is larger than iPSC expression. Summary table with results from differential expression analysis of transcripts that were up-regulated in MKs compared to iPSCs. The table includes the HGNC gene identifier “Gene” and the physical location of the transcript, given by chromosome, start and end position in genomic coordinates from genome assembly GRCh37/hg19. Parameter estimates of differences in MKs and IPSCs are given by log_2_ fold changes and corresponding fold changes. “p-value” and “q-value” show the statistical significance of differential expression before and after correction for multiple comparisons, respectively.
(XLSX)

S4 Table List of transcripts for which MK expression is smaller than iPSC expression. Summary table with results from differential expression analysis of transcripts that were down-regulated in MKs compared to iPSCs. The table includes the HGNC gene identifier “Gene” and the physical location of the transcript given by chromosome, start and end position in genomic coordinates from genome assembly GRCh37/hg19. Parameter estimates of differences in MKs and IPSCs are given by log_2_ fold changes and corresponding fold changes. “p-value” and “q-value” show the statistical significance of differential expression before and after correction for multiple comparisons, respectively.
(XLSX)

S1 Fig CNVs called by the hidden Markov model in iPSCs but not the corresponding donor DNA. (PDF)

S2 Fig CNVs called by the hidden Markov model in MKs but not the corresponding iPSC line. (PDF)

S3 Fig Five examples of CNVs present in the in donor DNA that are also present in the iPSCs and MKs. (PDF)

S4 Fig Principal component analysis (PCA) of 56 RNA-sequencing experiments. (PDF)

S5 Fig Differential Expression between iPSCs and MKs. (PDF)

S6 Fig Principal component analysis (PCA) by cell type and percent CD41+CD42a+ megakaryoblasts in MK pellet. (PDF)

S7 Fig Comparison of transcript expression filters. (PDF)
