# Supplementary material for: Integrity of Induced Pluripotent Stem Cell (iPSC) Derived Megakaryocytes as Assessed by Genetic and Transcriptomic Analysis
Source: PLoS One. 2017 Jan 20;12(1):e0167794. doi: 10.1371/journal.pone.0167794 (PMC5249236; doi:10.1371/journal.pone.0167794)
Supplement: S1 Table — Sources of DNA and RNA by cell type, passage number of iPSC lines and percent CD41+CD42a+ megakaryoblasts in MK pellets by study subject. (PDF) [file pone.0167794.s001.pdf]

S1 Table. Overview of study subjects.

Subject	Race	Age	Gender	Donor MNC	iPSC Line				MK Line		
				Genotype Array Data	Cell Line	Passage #	RNASeq Data	Genotype Array Data	Cell Line	CD41a/42a	RNASeq Data
P002	AA	63.0883	F	✔	IP002A	17	✔	✔	MP002A	65	✔
					IP002B	17	✔	✔	MP002B	81	✔
P003	EA	57.18001	M		IP003A	18	✔	✔	MP003A	93	✔
					IP003B	18	✔	✔	MP003B	80	✔
P005	AA	59.04449	M	✔	IP005A	6	✔		MP005A	80	✔
					IP005B	6	✔		MP005B	86	✔
P007	AA	60.98015	F		IP007A	5	✔		MP007A	90	✔
					IP007B	5	✔		MP007B	87	✔
P008	AA	61.74949	M		IP008A	8	✔		MP008A	90	✔
					IP008B	8	✔		MP008B	67	✔
P009	AA	50.36824	F		IP009A	6	✔		MP009A	87	✔
					IP009B	6	✔		MP009B	91	✔
P010	AA	75.06366	F		IP010A	3	✔		MP010A	87	✔
					IP010B	3	✔		MP010B	87	✔
P011	AA	70.10267	M		IP011A	4	✔		MP011A	81	✔
					IP011B	4	✔		MP011B	78	✔
P025	AA	47.14853	F	✔	IP025A	7		✔	MP025A	93	
					IP025B	7		✔	MP025B	75	
P026	AA	59.56468	F	✔	IP026A	13	✔	✔	MP026A	94	✔
					IP026B	13	✔		MP026B	88	✔
P028	AA	35.34565	F	✔	IP028A	9	✔	✔	MP028A	80	✔
					IP028B	9	✔	✔	MP028B	88	✔
P030	AA	36.59411	M	✔	IP030A	8	✔	✔	MP030A	66	✔
					IP030B	8	✔	✔	MP030B	66	✔
P061	EA	39.43874	M		IP061A	9	✔		MP061A	54	✔
					IP061B	11	✔		MP061B	47	✔
P068	EA	33.18823	M		IP068A	4	✔		MP068A	45	✔
					IP068B	4	✔		MP068B	46	✔
P069	EA	75.37029	F		IP069A	4	✔		MP069A	90	✔
					IP069B	4	✔		MP069B	89	✔
