# Supplementary material for: Integrity of Induced Pluripotent Stem Cell (iPSC) Derived Megakaryocytes as Assessed by Genetic and Transcriptomic Analysis
Source: PLoS One. 2017 Jan 20;12(1):e0167794. doi: 10.1371/journal.pone.0167794 (PMC5249236; doi:10.1371/journal.pone.0167794)
Supplement: S2 Table — Counts of discordant genotypes out of a total of 946,674 SNPs between pairs of lines within 6 GeneSTAR subjects. All samples had genotype data on DNA from the single donor MNCs, and the first line (A line) of iPSC with its corresponding derived MK. Five samples had a second line (B line) of iPSC and one sample also had the corresponding MK. Transmitted discordancies represent the discordant genotypes that are observed in the donor MNC-iPSC that is noted to be transmitted on to the corresponding derived MK. (PDF) [file pone.0167794.s002.pdf]

S2 Table. Genotype discordances.

Subject	A line pair		B line pair		‘transmitted’ discordancies
	MNC - iPSC	iPSC - MK	MNC - iPSC	iPSC- MK	
P002	4	5	4	n/a	0
P003	4	2	1	n/a	3
P025	87	112	73	n/a	8
P026	2	10	n/a	n/a	1
P028	6	4	3	n/a	2
P030	4	8	7	3	2-3
