# Supplementary material for: Integrity of Induced Pluripotent Stem Cell (iPSC) Derived Megakaryocytes as Assessed by Genetic and Transcriptomic Analysis
Source: PLoS One. 2017 Jan 20;12(1):e0167794. doi: 10.1371/journal.pone.0167794 (PMC5249236; doi:10.1371/journal.pone.0167794)
Supplement: S1 Fig — (PDF) [file pone.0167794.s005.pdf]

**S1 Fig. CNVs called by the hidden Markov model in iPSCs but not the corresponding donor DNA.**

In all instances, the log R ratios (LRRs) and B allele frequencies (BAFs) are qualitatively the same for the respective cells, indicating either a false positive call in the iPSC or a false negative call in the donor DNA. The colored dots indicate the called CNV (blue = duplication, red=deletion), the left two panels reference the LRR and BAF in the donor MNC and the right two panels reference the LRR and BAF in the iPSC line). Each horizontal row represents a different genomic region in which a CNV was called.

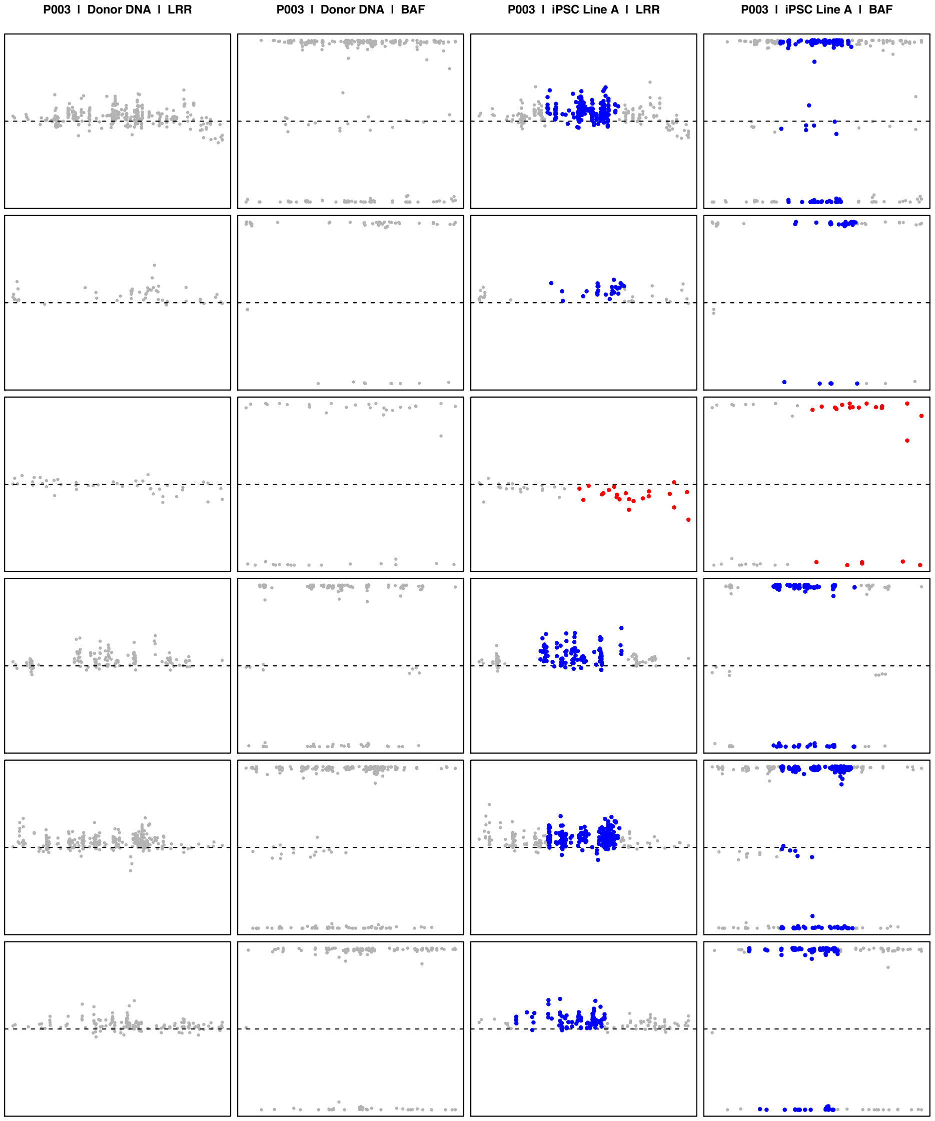


Example 1: CNVs called by the CNV algorithm in the iPSC A line for subject P003 and not in the donor DNA. However no qualitative differences are noted in the LRR and BAF between the donor and iPSC upon manual examination.

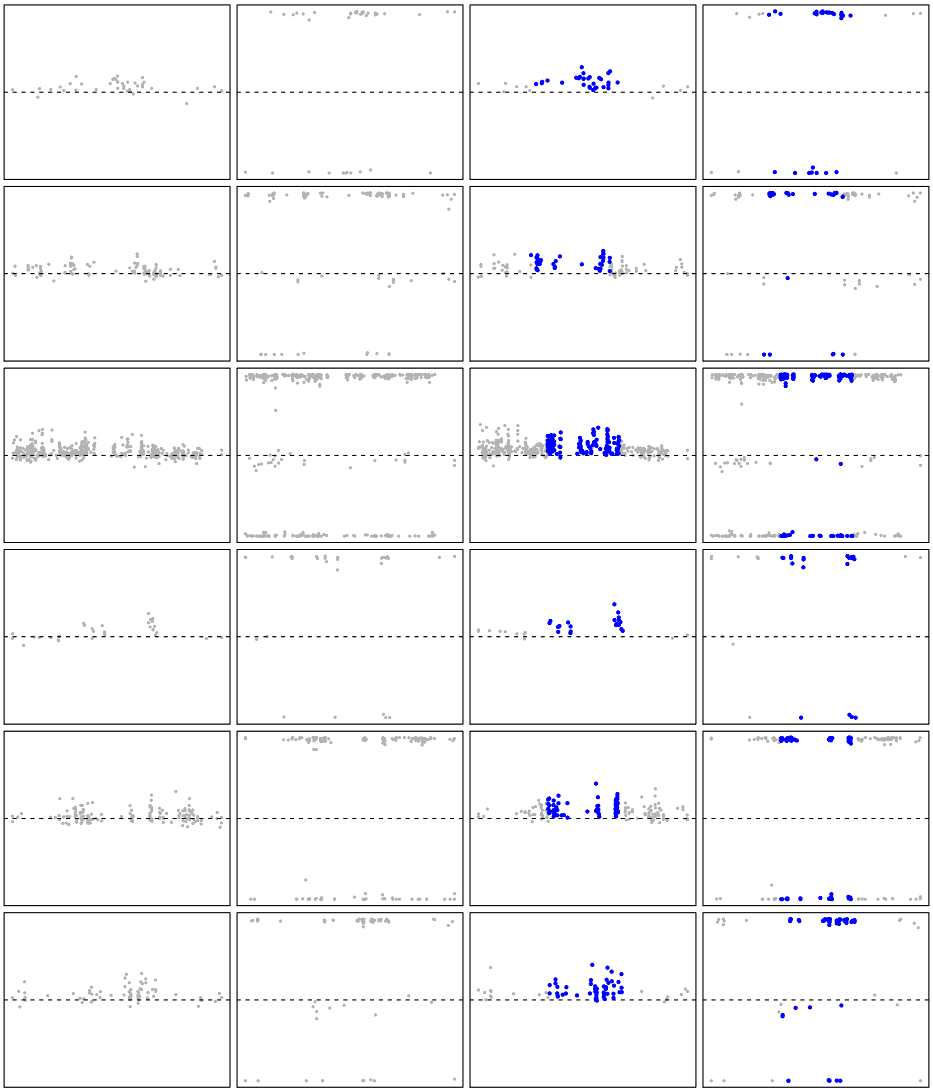

Example 1 continued: CNVs called by the CNV algorithm in the iPSC A line for subject P003 and not in the donor DNA. However no qualitative differences are noted in the LRR and BAF between the donor and iPSC upon manual examination.

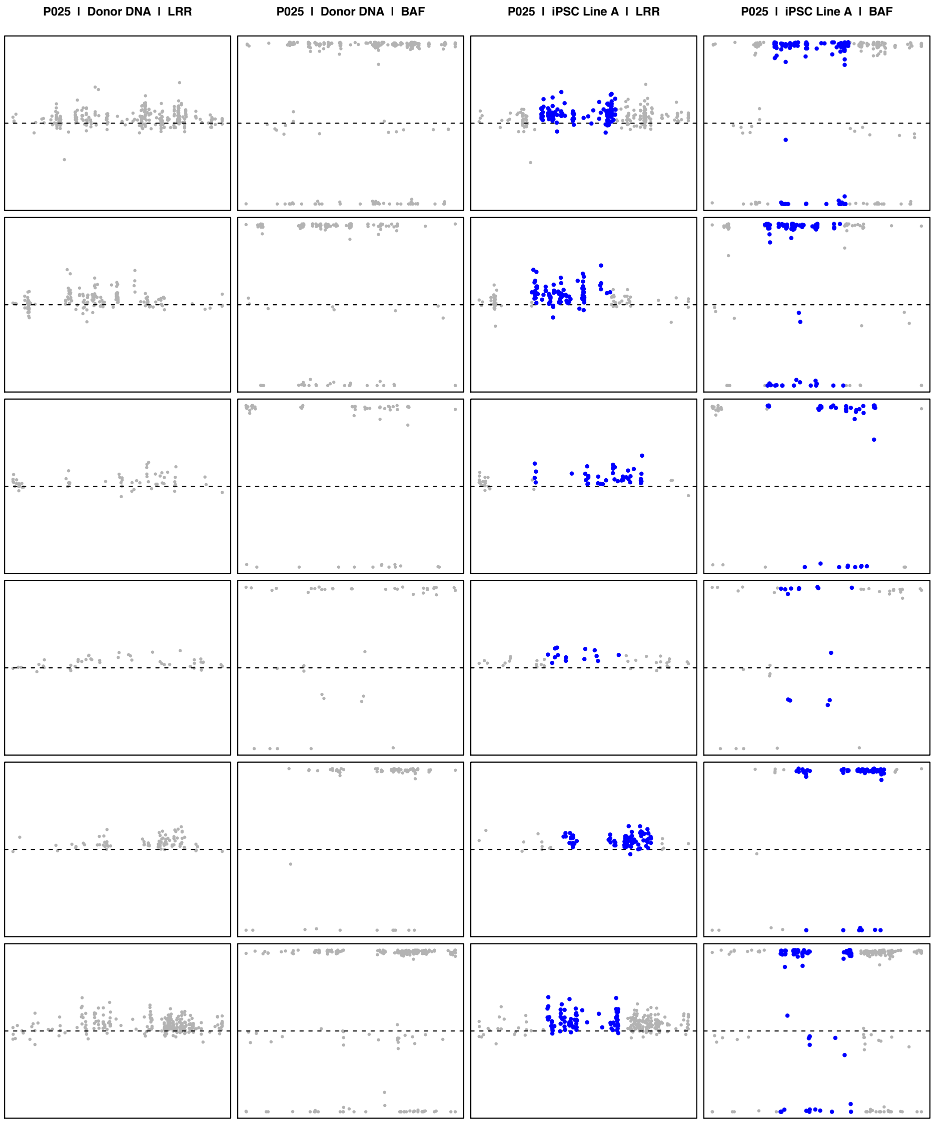


Example 2: CNVs called by the CNV algorithm in the iPSC A line for subject P025 and not in the donor DNA. However no qualitative differences are noted in the LRR and BAF between the donor and iPSC upon manual examination.

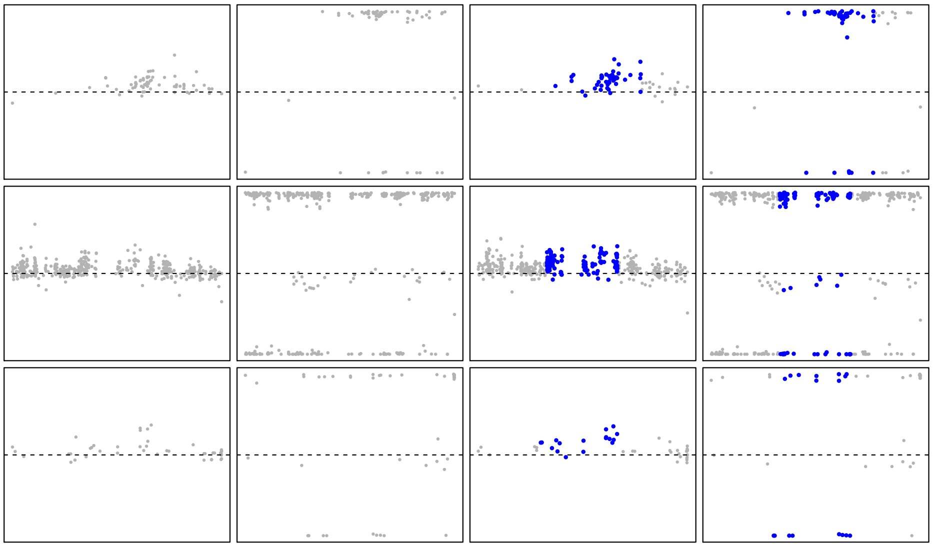

Example 2 continued: CNVs called by the CNV algorithm in the iPSC A line for subject P025 and not in the donor DNA. However no qualitative differences are noted in the LRR and BAF between the donor and iPSC upon manual examination.

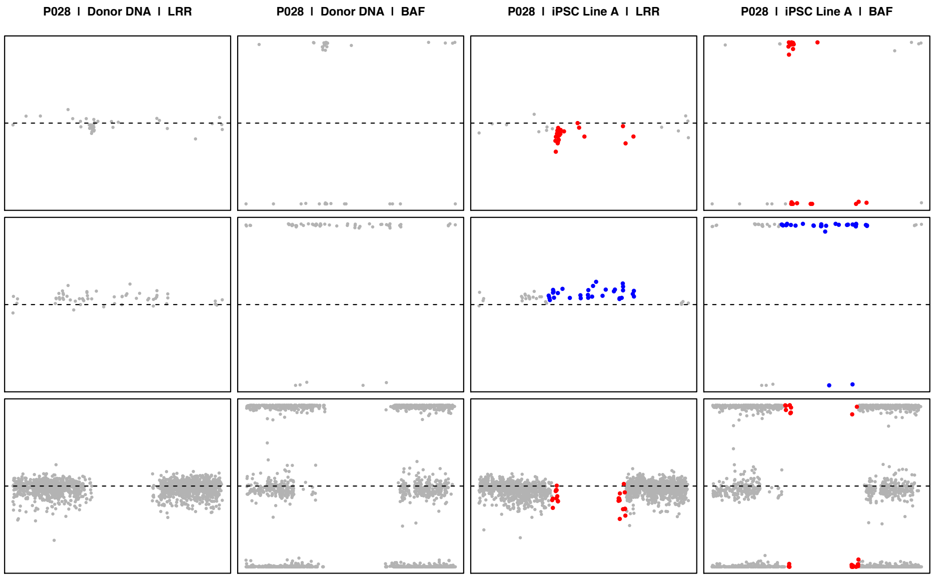


Example 3: CNVs called by the CNV algorithm in the iPSC A line for subject P028 and not in the donor DNA. However no qualitative differences are noted in the LRR and BAF between the donor and iPSC upon manual examination.

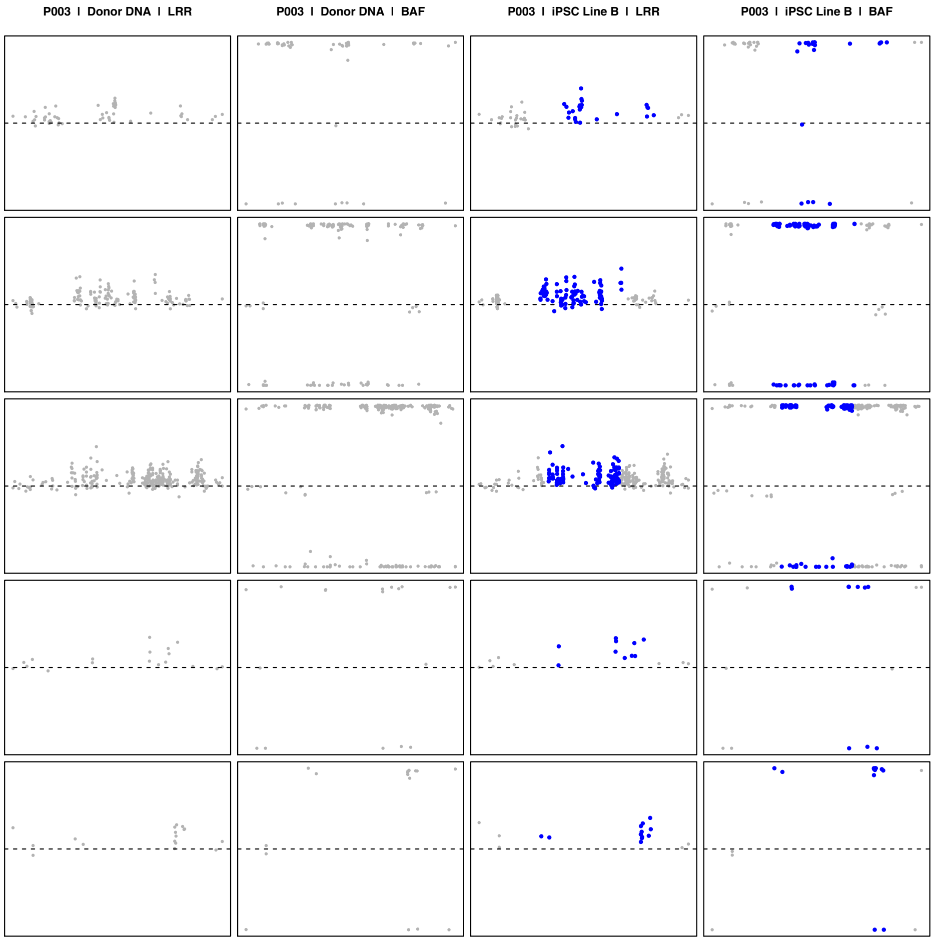


Example 4: CNVs called by the CNV algorithm in the iPSC B line for subject P003 and not in the donor DNA. However no qualitative differences are noted in the LRR and BAF between the donor and iPSC upon manual examination.

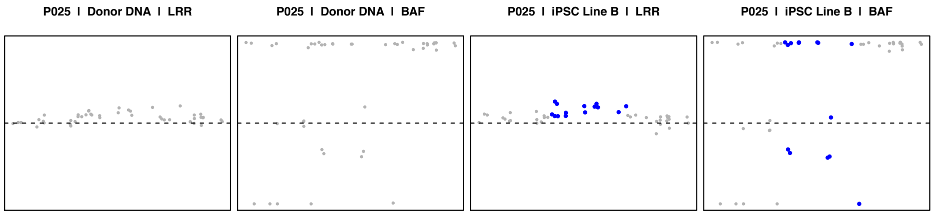


Example 5: CNVs called by the CNV algorithm in the iPSC B line for subject P025 and not in the donor DNA. However no qualitative differences are noted in the LRR and BAF between the donor and iPSC upon manual examination.

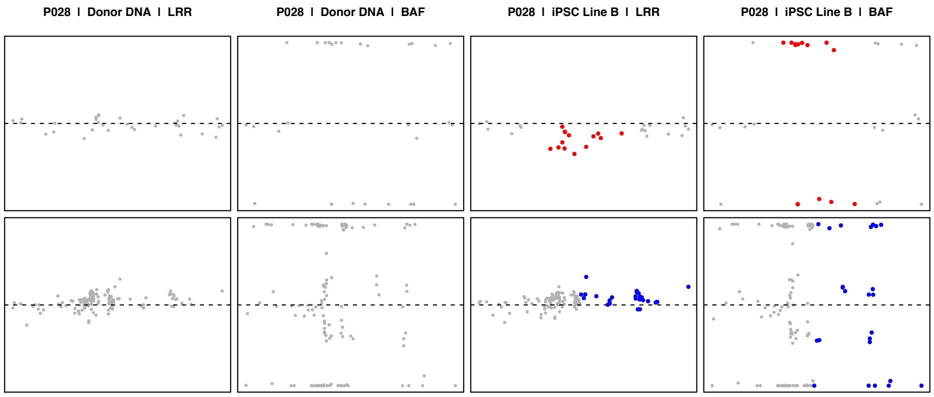


Example 6: CNVs called by the CNV algorithm in the iPSC B line for subject P028 and not in the donor DNA. However no qualitative differences are noted in the LRR and BAF between the donor and iPSC upon manual examination.
