# Supplementary material for: Integrity of Induced Pluripotent Stem Cell (iPSC) Derived Megakaryocytes as Assessed by Genetic and Transcriptomic Analysis
Source: PLoS One. 2017 Jan 20;12(1):e0167794. doi: 10.1371/journal.pone.0167794 (PMC5249236; doi:10.1371/journal.pone.0167794)
Supplement: S2 Fig — (PDF) [file pone.0167794.s006.pdf]

S2 Fig. CNVs called by the hidden Markov model in MKs but not the corresponding iPSC line.

In all instances, the log R ratios (LRRs) and B allele frequencies (BAFs) are qualitatively the same for the respective cells, indicating either a false positive call in the MK or a false negative call in the iPSC. The colored dots indicate the called CNV (blue = duplication, red=deletion), the left two panels reference the LRR and BAF in the donor MNC and the right two panels reference the LRR and BAF in the iPSC line). Each horizontal row represents a different genomic region in which a CNV was called.

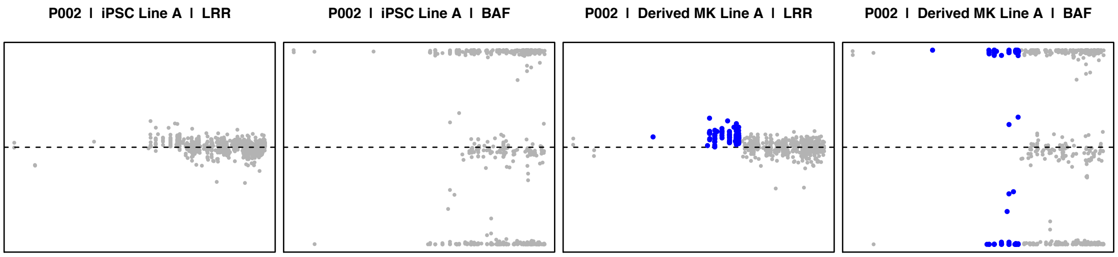

Example 1: CNVs called by the CNV algorithm in the MK A line for subject P002 and not in the iPSC A line. However no qualitative differences are noted in the LRR and BAF between the MK and iPSC lines upon manual examination.

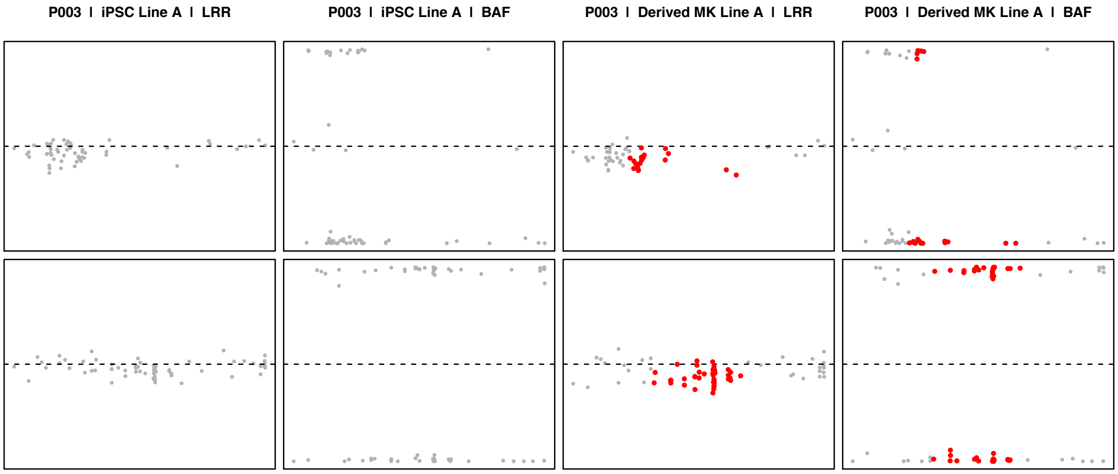

Example 2: CNVs called by the CNV algorithm in the MK A line for subject P003 and not in the iPSC A line. However no qualitative differences are noted in the LRR and BAF between the MK and iPSC lines upon manual examination.

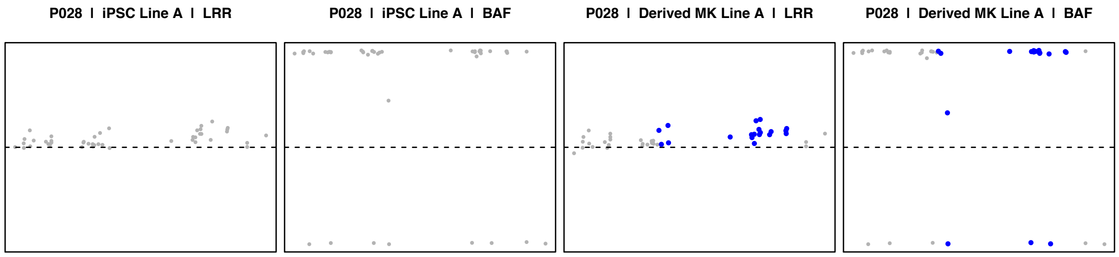

Example 3: CNVs called by the CNV algorithm in the MK A line for subject P028 and not in the iPSC A line. However no qualitative differences are noted in the LRR and BAF between the MK and iPSC lines upon manual examination.

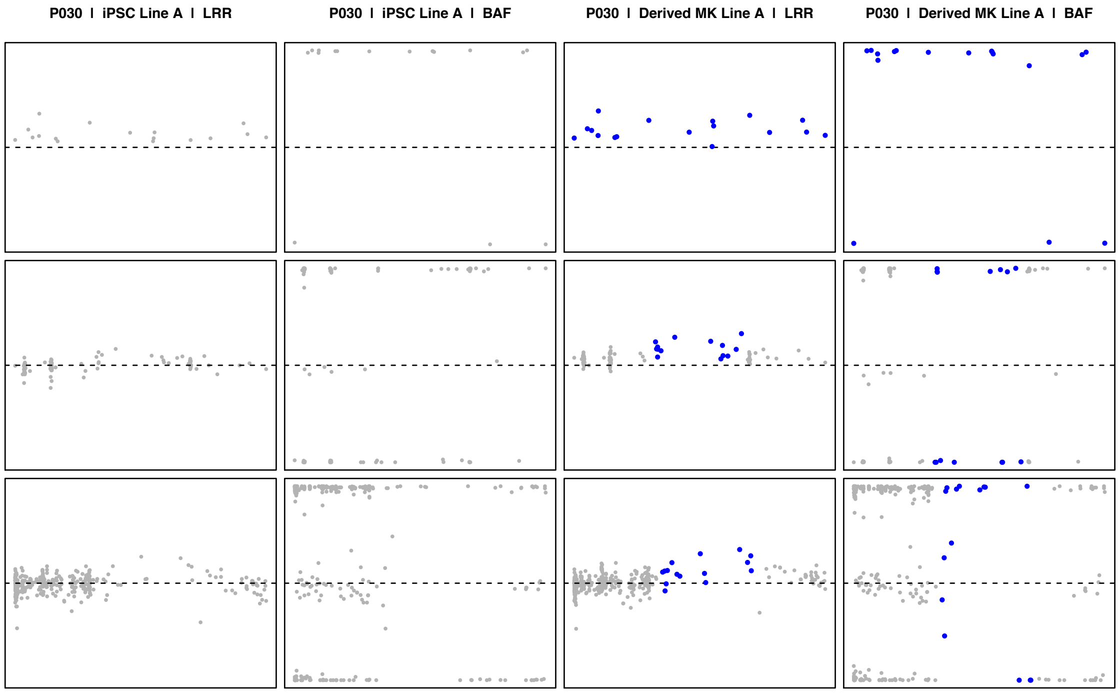

Example 4: CNVs called by the CNV algorithm in the MK A line for subject P030 and not in the iPSC A line. However no qualitative differences are noted in the LRR and BAF between the MK and iPSC lines upon manual examination.

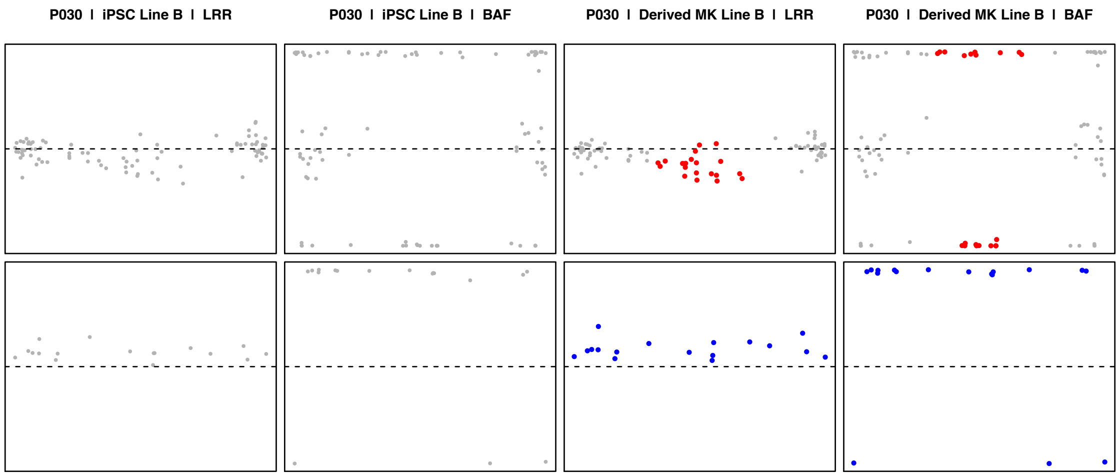

Example 5: CNVs called by the CNV algorithm in the MK B line for subject P030 and not in the iPSC B line. However no qualitative differences are noted in the LRR and BAF between the MK and iPSC lines upon manual examination.
