# Supplementary material for: Integrity of Induced Pluripotent Stem Cell (iPSC) Derived Megakaryocytes as Assessed by Genetic and Transcriptomic Analysis
Source: PLoS One. 2017 Jan 20;12(1):e0167794. doi: 10.1371/journal.pone.0167794 (PMC5249236; doi:10.1371/journal.pone.0167794)
Supplement: S3 Fig — (PDF) [file pone.0167794.s007.pdf]

S3 Fig. Five examples of CNVs present in the in donor DNA that are also present in the iPSCs and MKs.

Red dots represent deletions, and blue dots represent duplications.

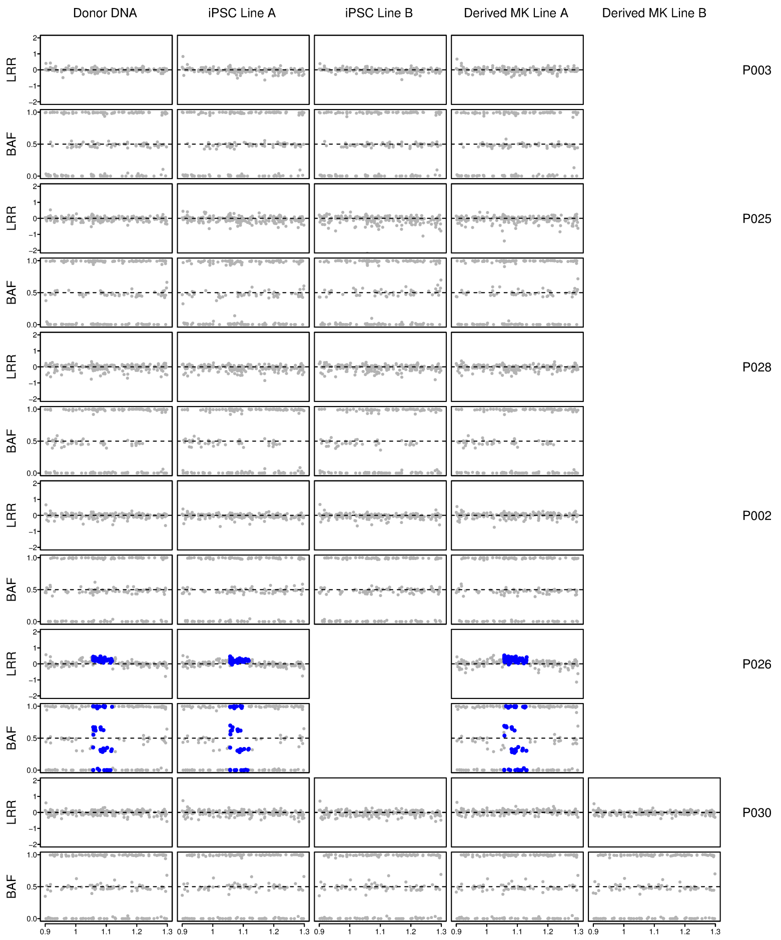

Example 1: A duplication (three copies, blue dots) in P026 on chromosome 9 near locus 1.1Mb.

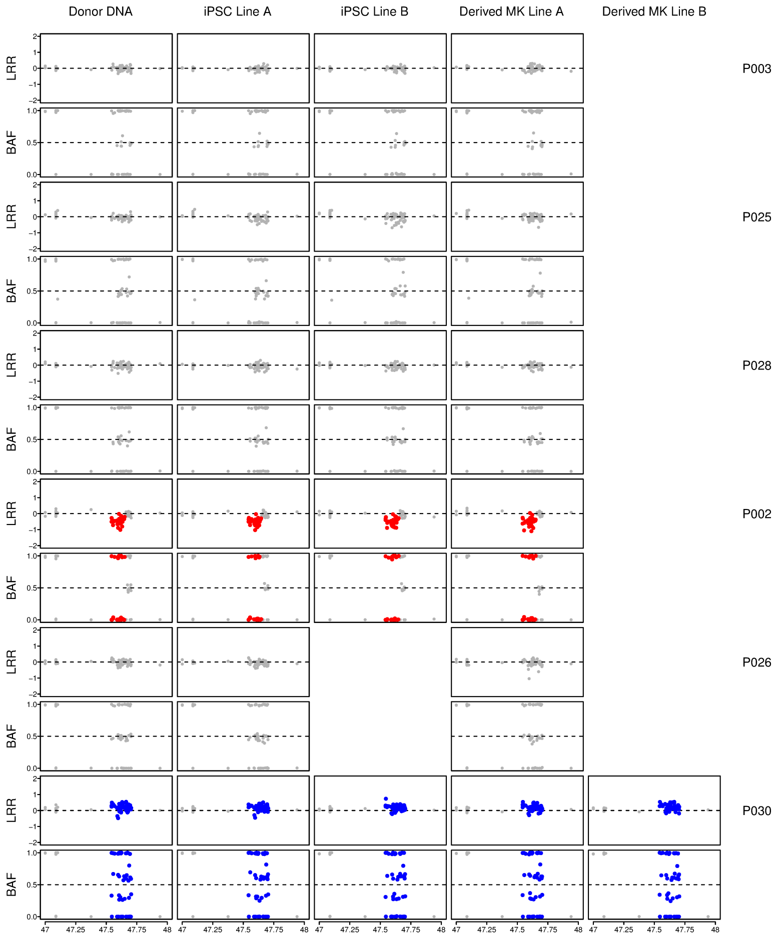

Example 2: A potential copy number polymorphism on chromosome 10 near locus 47.6Mb. Hemizygous deletions are observed in the P002 cells (red dots) and duplications (blue dots) are observed in the P030 cells.

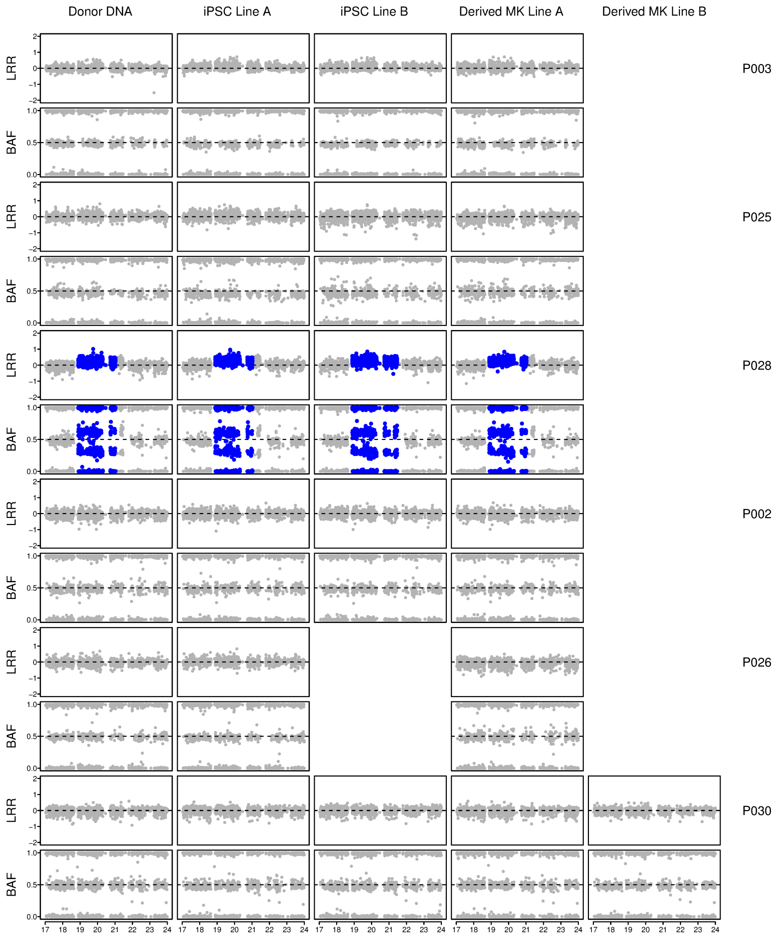

Example 3: A duplication (three copies, blue dots) in P028 on chromosome 22 near locus 20.2Mb.

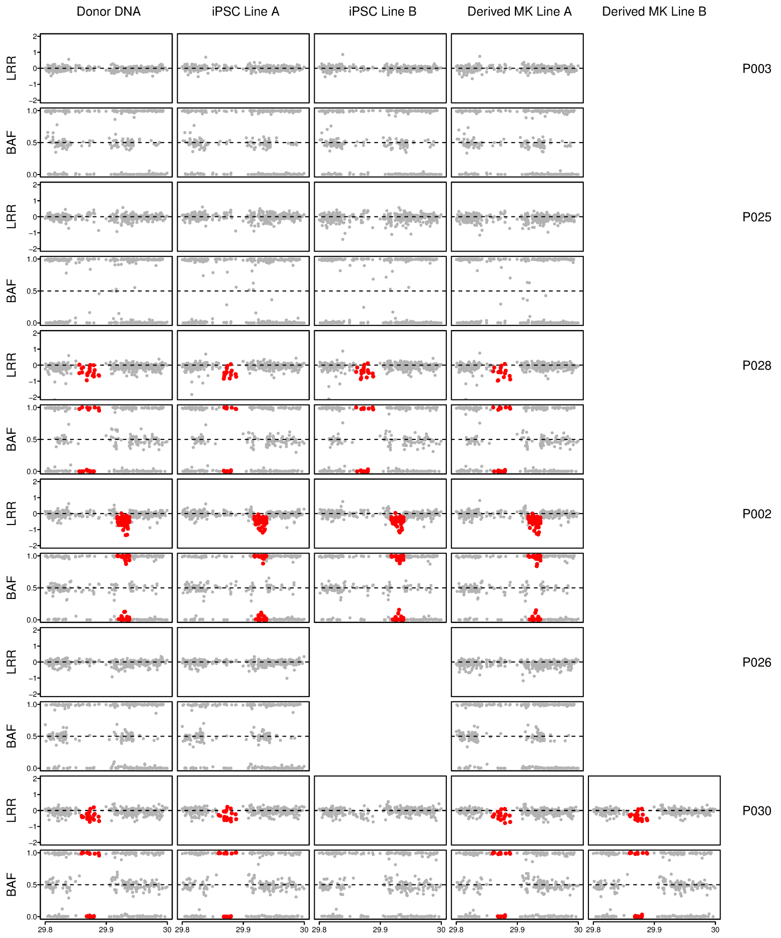

Example 4: A deletion on chromosome 6 (red dots, left of 29.9Mb) called in P002 and P030, except for the P030 iPSC Line B. The deletion calls overall are somewhat ambiguous, and the deletion cannot be inferred with complete confidence. Note that the data in P030 iPSC Line B are qualitatively the same as in the other cells.

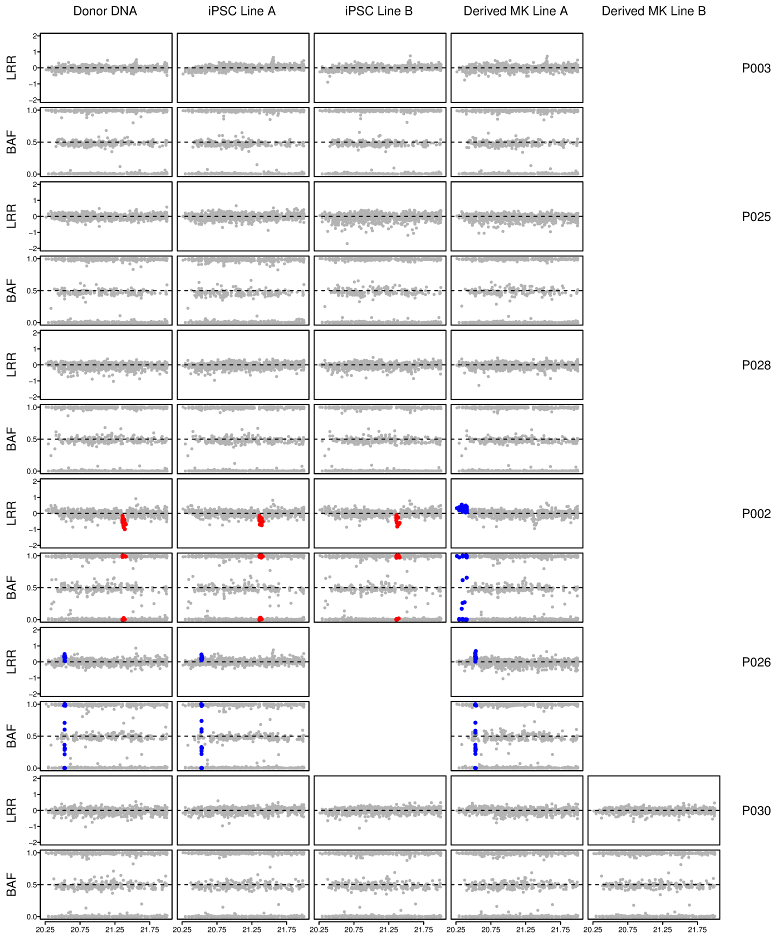

Example 5: A deletion (red dots) and duplications (blue dots) on chromosome 14 near 20.7Mb. The amplification in P026 has been consistently called. The deletion in P002 was missed by the calling algorithm in the MK Line A but is qualitatively the same as in the other cells for P002. On the other hand, the duplication called in P002 MK Line A was missed in the other P002 cells, but clearly is present.
