# Supplementary material for: Integrity of Induced Pluripotent Stem Cell (iPSC) Derived Megakaryocytes as Assessed by Genetic and Transcriptomic Analysis
Source: PLoS One. 2017 Jan 20;12(1):e0167794. doi: 10.1371/journal.pone.0167794 (PMC5249236; doi:10.1371/journal.pone.0167794)

S4 Fig. Principal component analysis (PCA) of 56 RNA-sequencing experiments.
PCA analysis reveals that the first PC accounts for 47.1% and the second PC for 13.0% of the variation observed in 56 RNA-sequencing experiments representing 14 independent subjects each with technical replicates of paired iPSC-MK lines.

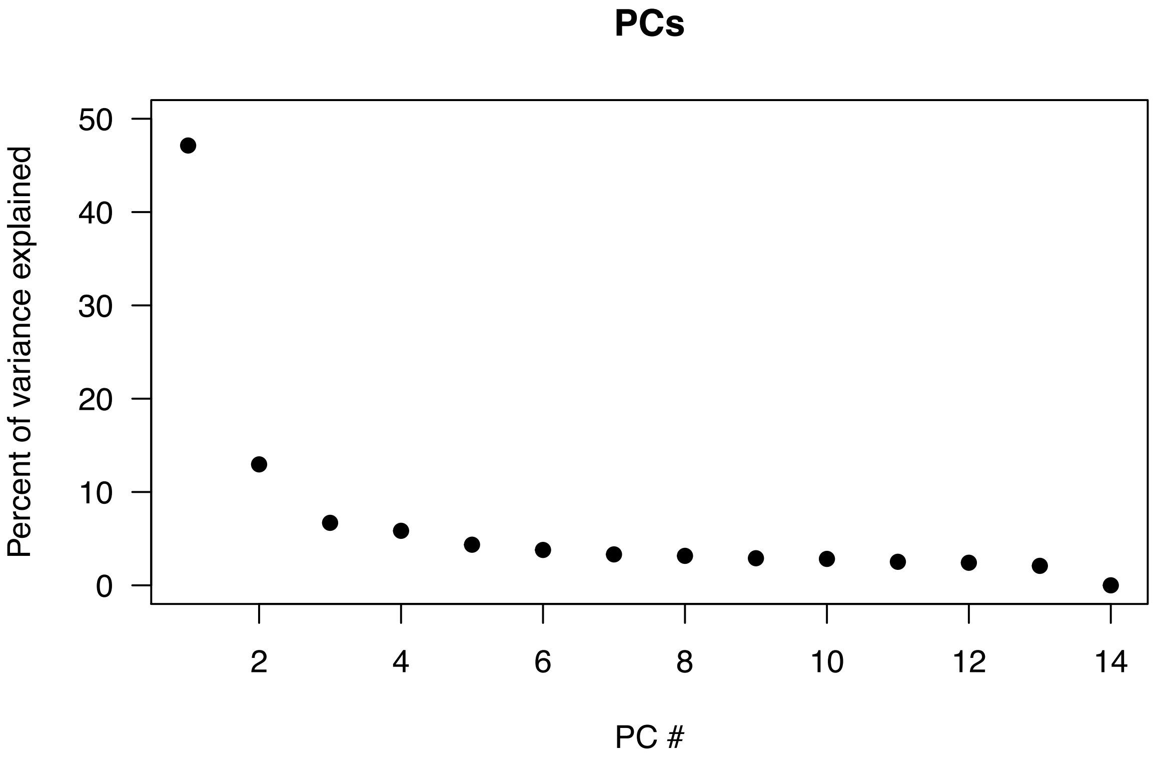

Supplement: S4 Fig — (PDF) [file pone.0167794.s008.pdf]
