# Supplementary material for: Integrity of Induced Pluripotent Stem Cell (iPSC) Derived Megakaryocytes as Assessed by Genetic and Transcriptomic Analysis
Source: PLoS One. 2017 Jan 20;12(1):e0167794. doi: 10.1371/journal.pone.0167794 (PMC5249236; doi:10.1371/journal.pone.0167794)

S5 Fig. Differential Expression between iPSCs and MKs.
Inference for 14 independent subjects each with technical replicates of paired iPSC-MK lines and 33,287 transcripts. The volcano plot shows the estimated $\log_2$ fold changes (x-axis) versus the $-\log_{10}$ p-values (y-axis) for each transcript (left panel). Histograms show the distributions of p-values from differential expression tests for transcripts up-regulated in MKs compared to iPSCs (top right panel) and transcripts down-regulated in MKs compared to iPSCs (bottom right panel). The majority of transcripts show statistically significant differences in expression between the two cell types.

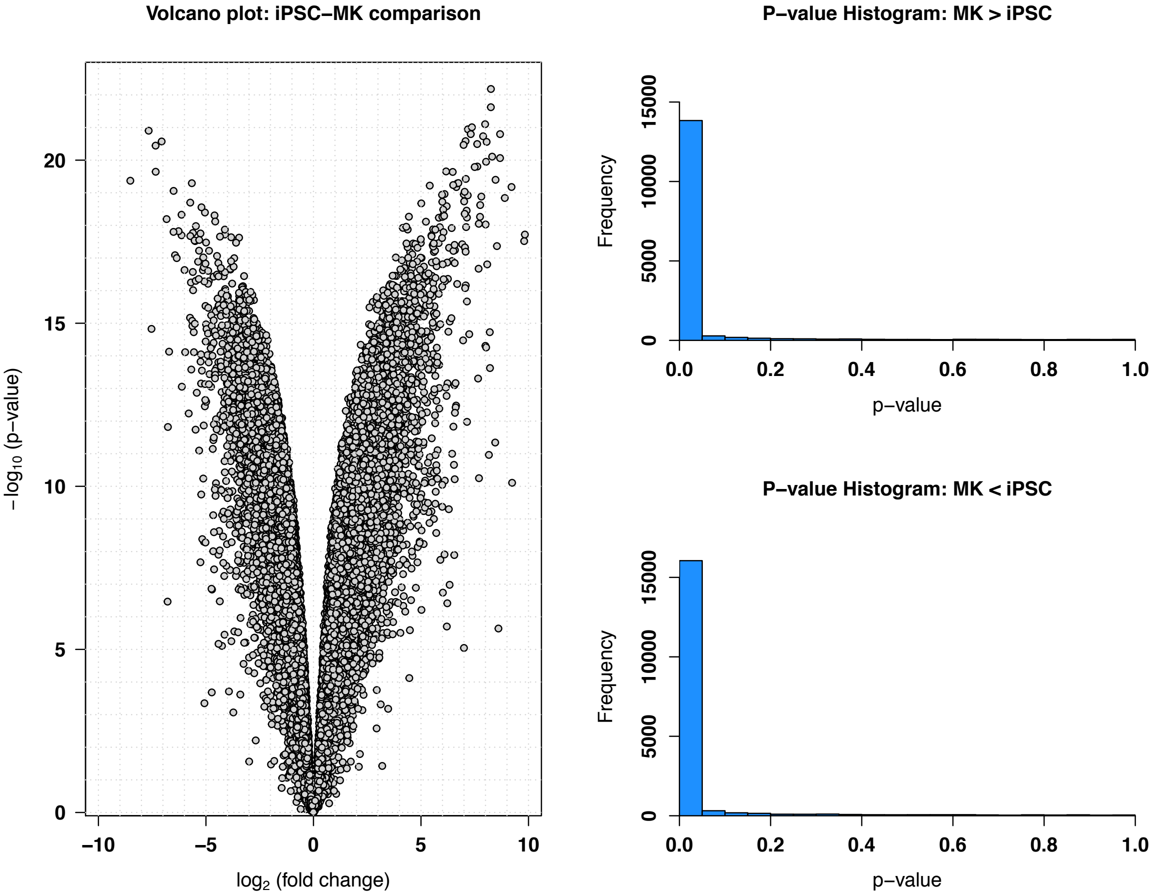

Supplement: S5 Fig — (PDF) [file pone.0167794.s009.pdf]
