# Supplementary material for: Integrity of Induced Pluripotent Stem Cell (iPSC) Derived Megakaryocytes as Assessed by Genetic and Transcriptomic Analysis
Source: PLoS One. 2017 Jan 20;12(1):e0167794. doi: 10.1371/journal.pone.0167794 (PMC5249236; doi:10.1371/journal.pone.0167794)

S6 Fig. Principal component analysis (PCA) by cell type and percent $CD41^+CD42a^+$ megakaryoblasts in MK pellet.
Scatter plot showing the relationship between cell types in terms of principal component 1 (x-axis) and principal component 2 (y-axis) from a PCA of 33,287 transcripts with FPKM interquartile range larger than 1. Transparency of color for the MK cells corresponds to the percentage of the $CD41^+CD42a^+$ megakaryoblasts in MK pellet. Darker orange colors indicate higher $CD41^+CD42a^+$ percentages as indicated by the legend and actual values are given in numbers next to the sample. We do not detect any apparent pattern or cluster of cells with lower or higher $CD41^+CD42a^+$ values.

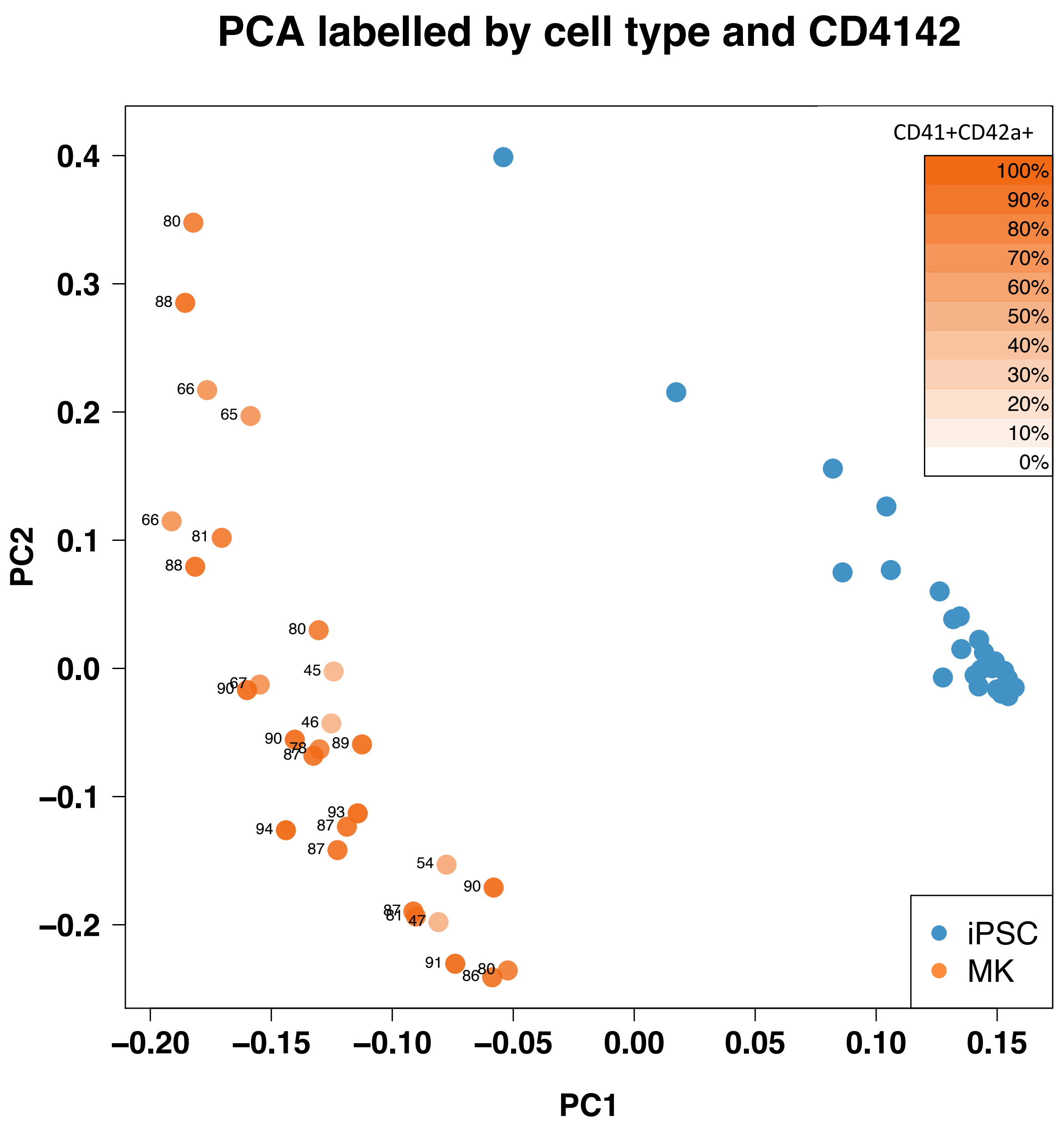

Supplement: S6 Fig — (PDF) [file pone.0167794.s010.pdf]
