# Supplementary material for: Integrity of Induced Pluripotent Stem Cell (iPSC) Derived Megakaryocytes as Assessed by Genetic and Transcriptomic Analysis
Source: PLoS One. 2017 Jan 20;12(1):e0167794. doi: 10.1371/journal.pone.0167794 (PMC5249236; doi:10.1371/journal.pone.0167794)

S7 Fig. Comparison of transcript expression filters.
The four panels show the expression measurements (on FPKM scale) for four selected transcripts that show different expression patterns across 56 samples in total, separated into 28 iPSCs (blue) and 28 MKs (orange). For each transcript the median, the variance and the interquartile range (IQR) across all 56 samples are calculated and displayed. The cutoff for inclusion of a transcript into a filtered data set is set to 1 for each potential filter. “Transcript 1” reveals overall very low expression for both cell types and is thus excluded by all filtering methods. “Transcript 2” shows very low expression for iPSCs and high expression for MKs. This transcript is kept independent of the filter choice. Similar to “Transcript 2”, “Transcript 3” shows very low expression for iPSCs and moderate expression in MKs. This transcript would be excluded with the median filter and kept by the variance and IQR filter. In “Transcript 4” the overall expression values are very low except for one outlier sample in the MKs. This results in exclusion of this transcript by the median and IQR filters, but inclusion by the variance filtering method. Transcript 3 highlights the importance of our choice of the IQR filter as in our experimental design it is of interest to retain transcripts that may be essentially 0 in one cell type and low in another.

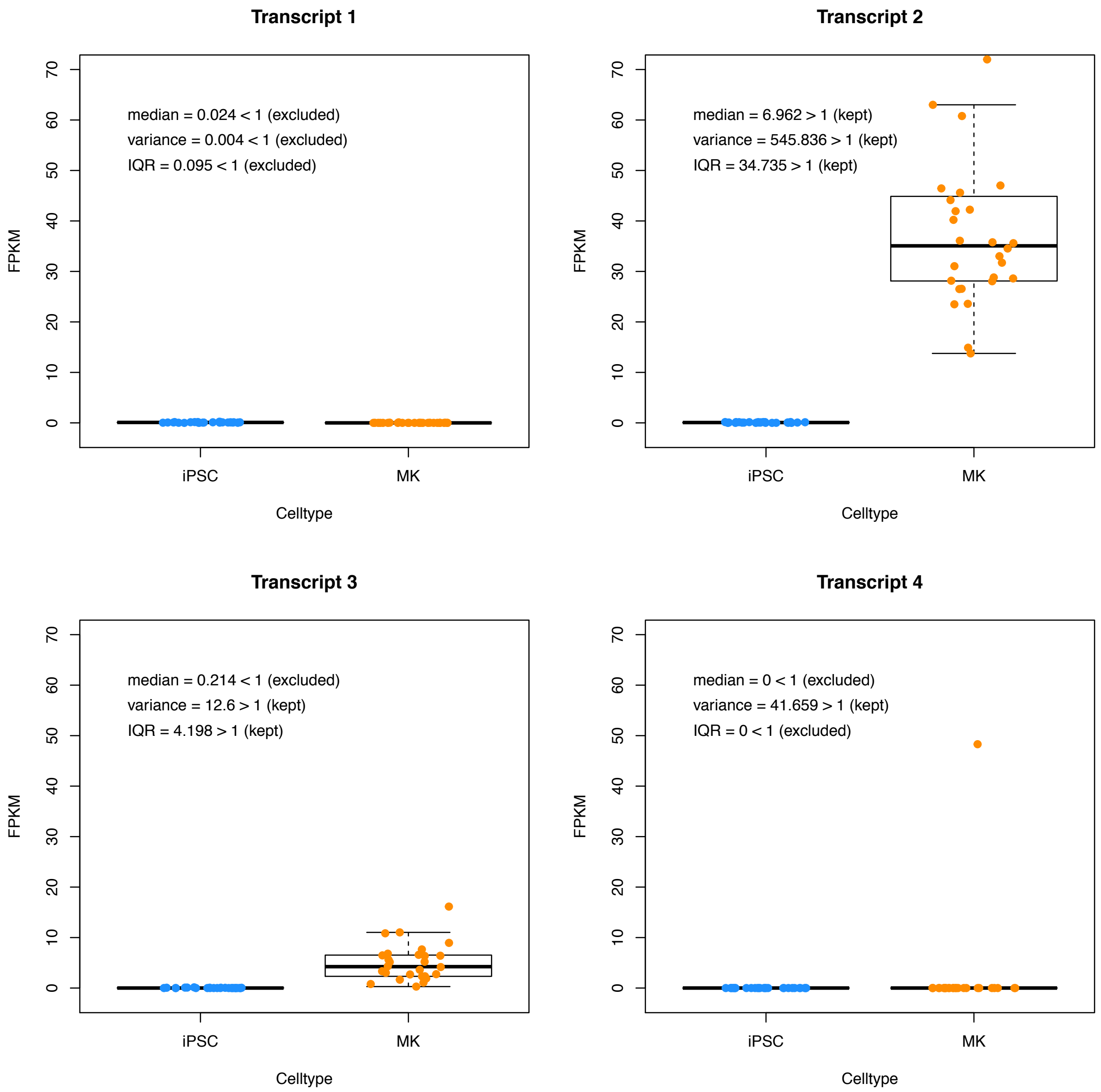

Supplement: S7 Fig — (PDF) [file pone.0167794.s011.pdf]
